# Some Aspects of Intestinal Obstruction

**Published:** 1908-07-18

**Authors:** 


### SOME ASPECTS OF INTESTINAL OBSTRUCTION.

### THE HISTORY OF A TYPICAL CASE OF CHRONIC OBSTRUCTION.

The onset of symptoms in a typical case of obstruction due to a progressive stenosis is in the great majority of cases gradual, and a patient may be afflicted with intestinal stenosis for many weeks, or even months, before he is himself aware that he is unwell. His attention is first called to his condition by a sense of increasing discomfort in the abdomen, accompanied by eructation and other symptoms of disturbance of his digestive function. At the same time he begins to notice that he is constipated.

In some cases the stenosis increases without causing symptoms until the lumen of the gut is extremely narrowed. This happens more easily in the small intestine, where the intestinal contents are liquid, than in the colon, where the fæces are beginning to assume their formed character. At this stage some undigested residue of food material or a foreign body may reach the stricture, and, being unable to go through, may lead to an occlusion of the gut. Where this happens the onset of symptoms is of an acute nature, and often leads to a mistaken diagnosis.

The difference in the condition of the contents of the small and large intestine explains the fact that constipation is a more marked feature when the stenosis occurs low down in the alimentary canal. This rule, however, like most other rules of surgery, must not be regarded as absolute. Nothnagel quotes a case of carcinoma of the sigmoid where the patient had a daily evacuation of the bowels up to ten days before her death.

The constipation gradually becomes more and more severe and necessitates the use of purges, which in time lose their power of moving on the contents of the bowel, so that the strength of the aperient used is progressively increased. It is generally varied at this period by occasional attacks of diarrhœa in which very large quantities of loose fæcal matter and gas are evacuated, with great relief to the patient. The diarrhœa is a spurious diarrhœa. The bowel above the stricture is hypertrophied, and its blood-vessels become engorged, while its mucous membrane is irritated by the presence of long-retained products of decomposition and congested by reason of the distension of the bowel wall. There results a catarrh of the mucous membrane above the stricture which leads to a copious watery discharge, and this fluid, carrying with it a certain amount of suspended fæcal matter, escapes at the anus as a loose evacuation. The motions thus passed are intensely foul. The significance of this syndrome, constipation alternating with diarrhœa, can hardly be exaggerated, and every case which presents these features should be suspected of incipient chronic intestinal obstruction.

Paroxysmal attacks of colicky pain supervene to add to the patient's distress. At first they are not very severe, pass off shortly, and only recur at considerable intervals of time, but they tend to increase in frequency, intensity, and duration. In some cases the colic is localised to the area of the stenosis, but it is more often diffuse. In rare cases it is localised to a part of the abdomen other than the seat of the disease. As the disease progresses the pain of the paroxysms becomes excruciating, and the interval between the attacks is so much reduced that it becomes practically continuous. When this occurs the patient presents the symptom-complex of complete intestinal occlusion.

The paroxysms of pain are associated with, and caused by, increased peristalsis of the coils of intestine. In all cases of chronic obstruction of some standing energetic contractions of the intestine can be seen through the parietes, and the surface of the abdomen has been very aptly compared to "a relief map of a hilly country."

It must be remembered that physiological peristalsis of the intestinal coils may be visible in very thin patients, but in these cases it is quite sluggish. In chronic obstruction the movements are most energetic. The intestines can be seen to stiffen and contract in the effort to overcome the obstruction. The coils are never visible in the same place for any length of time, although the same series of phenomena will be frequently repeated in the same area of the abdominal wall—*e.g.* a coil will be seen to stiffen, to become peristaltic, and to remain in tetanic contraction for some seconds and then fade away. A small external stimulus, such as flicking or tapping the abdomen with the finger, is usually sufficient to call forth a repetition of the phenomenon.

This visible peristalsis (which is above all other symptoms pathognomonic of chronic intestinal obstruction) is the outward expression of certain anatomical changes which may well be considered at this point. In any form of chronic stenosis the intestine below the stenotic area is empty and contracted, but in all other respects normal (this is the *hungerdarm* of German writers). Above the stenosis there is found a series of changes largely produced by a stagnation of bowel contents. The stagnant contents cause distension, which induces increased activity in the bowel wall, and, as a result, the muscular coat becomes hypertrophied in the same way as any other muscle which is called on to do an abnormal amount of work. Microscopically it can be shown that this is a true hypertrophy, and not a hyperplasia—that is, the muscle cells are enlarged individually, but their number is not increased. Herezel found that this hypertrophy can be produced experimentally in animals by partial ligature of the alimentary canal. It is recognisable histologically upon the fourth day, and macroscopically upon the ninth. During the early stages the hypertrophy overcomes the stenosis, but only for a time, for sooner or later the hypertrophied muscle will become fatigued and paretic so that it can no longer drive on the intestinal contents, just as the muscular coat of the bladder is first hypertrophied behind a stricture but eventually becomes atonic, so that acute retention of urine ensues. It is this that explains the very thin, apparently membranous, and non-muscular, wall of the intestine as seen during operations for acute obstruction.

